# Severity-Dependent Long-Term Post-Traumatic Changes in the Circulating Oxylipin Profile

**DOI:** 10.3390/ijms252413530

**Published:** 2024-12-17

**Authors:** Madlen Reinicke, Leyu Zheng, Moujie Rang, Carolin Fuchs, Juliane Weikert, Annette Keß, Christian Kleber, Uta Ceglarek, Georg Osterhoff, Gabriela Aust

**Affiliations:** 1 Institute of Laboratory Medicine, Clinical Chemistry and Molecular Diagnostics, University Hospital Leipzig, 04103 Leipzig, Germany; madlen.reinicke@medizin.uni-leipzig.de (M.R.); juliane.weikert@medizin.uni-leipzig.de (J.W.); uta.ceglarek@medizin.uni-leipzig.de (U.C.); 2 Research Laboratories and Clinic of Orthopedics, Trauma and Plastic Surgery, Leipzig University and University Hospital Leipzig, 04103 Leipzig, Germany; leyu.zheng@medizin.uni-leipzig.de (L.Z.); moujie.rang@medizin.uni-leipzig.de (M.R.); carolin.fuchs@medizin.uni-leipzig.de (C.F.); annette.kess@medizin.uni-leipzig.de (A.K.); christian.kleber@medizin.uni-leipzig.de (C.K.); georg.osterhoff@medizin.uni-leipzig.de (G.O.); 3 Leipzig Medical Biobank, Medical Faculty, Leipzig University, 04103 Leipzig, Germany; 4 Research Laboratories and Clinic of Visceral, Transplantation, Vascular and Thoracic Surgery, Leipzig University and University Hospital Leipzig, 04103 Leipzig, Germany

**Keywords:** polyunsaturated fatty acid, oxylipin, arachidonic acid, injury, polytrauma

## Abstract

Trauma causes the breakdown of membrane phospholipids and the subsequent degradation of the released polyunsaturated fatty acids (PUFAs) to partially bioactive oxylipins. Here, we screened for circulating PUFAs and oxylipins in patients (n = 34) differing from those of uninjured controls (n = 25) and analyzed their diagnostic potential. Patients were followed up for 1 to 240 h after minor/moderate, severe, and very severe injuries. Of the targeted oxylipins, 13 out of 80 (13/80) were detected in almost all patients and controls. Injury caused a long-term decrease in 9- and 13-hydroxyoctadecadienoic acids and in several dihydroxyeicosatetraenoic acids, the stable derivatives of bioactive anti-inflammatory epoxyeicosatrienoic acids, compared to controls. Frequently, these oxylipins correlated inversely to injury severity, days in the intensive care unit and hospital, and/or procalcitonin and pro-inflammatory cytokine levels 48 up to 240 h after trauma. Notably, 20/80 oxylipins were detected in some patients but not or less often in controls. Many of these oxylipins increased transiently immediately after injury. Their level is partly correlated with adverse clinical parameters at this early time point. The circulating oxylipidome was markedly affected by trauma. Several oxylipins showed injury-dependent alterations at different time points in the post-traumatic course.

## 1. Introduction

Severe injury results in damage of macro- and micro-barriers, thereby exposing the immune system to damage-associated molecular patterns (DAMPs), which rapidly induce intracellular signaling in immune cells, particularly neutrophilic granulocytes, via pattern recognition receptors (PRR) [1]. In consequence, activated immune cells release myriads of structurally and stereochemically distinct, partially bioactive oxylipins derived from polyunsaturated fatty acids (PUFAs) by oxygenation. PUFAs are vital components of cell membrane phospholipids in our body. Two, linoleic acid (LA; omega-6, ω6) and alpha-linolenic acid (ALA, omega-3, ω3), cannot be synthesized by humans and must be obtained from the diet. LA is metabolically converted to gamma-linolenic acid (GLA), dihomo-GLA (DHGLA), and finally to arachidonic acid (ARA) and ALA to eicosapentaenoic acid (EPA) or docosahexaenoic acid (DHA), respectively.

PRR signaling causally activates (cytosolic) phospholipases A2 (PLA_2_), which then translocate to perinuclear and endoplasmic reticulum membranes [2]. The PLA_2_s hydrolyze membrane phospholipids, resulting in the release of PUFAs that undergo further metabolism involving at least one step of dioxygen-dependent oxidation to form oxylipins. Their biosynthesis can either be enzymatic utilizing promiscuous cyclooxygenases (COXs), lipoxygenases (LOXs), and cytochrome P450 mixed-function oxidases (CYPs) or non-enzymatic via free radical mechanisms [3].

ARA-derived eicosanoids produced by COX-1/-2 are predominantly pro-inflammatory and include classical prostaglandins (PGs) and thromboxanes (Txs). Leukotrienes (LTs) and lipoxins (LXs) are mainly generated by the activity of several LOXs. Bioactive but unstable epoxyeicosatrienoic acids (EETs) and their stable derivatives dihydroxyeicosatrienoic acids (DHETs) are converted by the most upstream members of the CYP family. Within the various originating oxylipin families, not all of which are listed here, there are members that exhibit either pro-inflammatory or anti-inflammatory/inflammation pro-resolving activities, some of which are context and cell-type dependent [4,5]. Very recently, oxylipins released after pyroptosis, a type of cell death, have been identified to help tissue healing. The supernatants of pyroptotic bone-marrow-derived macrophages and CD14^+^ blood monocytes induced a sharp increase in the rate of fibroblast wound closure [6]. Surprisingly, the de novo-generated oxylipin PGE_2_ was identified as promoting this healing effect.

Despite the important role of circulating, partly bioactive oxylipins they have not been investigated long-term in injured patients. Overall, oxylipodomic profiling from fluids and tissues is challenging due to the low abundance, instability, and difficult separation of structurally similar metabolites [7]. Here, we applied targeted liquid chromatography-tandem mass spectrometry (LC-MS/MS)-based profiling of seven circulating PUFAs and 80 PUFA-derived metabolites [7,8,9]. Plasma was collected at 1, 8, 24, 48, 120, and 240 h post-injury to observe the immediate effects of trauma and to track long-term anti-inflammatory/pro-resolving mediators. We included trauma patients with a wide range of injury severity to observe the effects of minor/moderate to very severe trauma, compared with an uninjured control group, from which we obtained plasma once. Our aim was to determine whether oxylipins could serve as potential markers for monitoring the time course and outcome of injured patients.

## 2. Results

### 2.1. Patient Groups Differ in Their Clinico-Pathological and Laboratory Parameters

Patients were assigned to three categories according to their injury severity score (ISS): minor/moderately (ISS < 16, n = 11, group 1), severely (ISS 16–24, n = 11, group 2), and very severely injured (ISS ≥ 25, n = 14, group 3) [10]. The length of stay at the intensive care unit (ICU) and in the hospital, as well as the Sequential Organ Failure Assessment (SOFA) score at 24 h after trauma, differed between the minor/moderately and very severely injured patients (Table 1). The majority of the very severely injured patients suffered from thoracic and abdominal injuries, as well as spinal injuries (Table S1).

Heat maps visualize the relationship between the patient’s parameters and scores at 1 and 24 h after injury (Figure S1A,B). Overall, the expected correlations between ISS, days at ICU and in the hospital, the various trauma-related clinical scores such as the SOFA score, Acute Physiology and Chronic Health Evaluation II (APACHE II) score, Simplified Acute Physiology Score 3 (SAPS3), and the Glasgow Coma Scale (GCS), as well as procalcitonin (PCT) and pro-inflammatory cytokine levels were present in the traumatized patients. Compared to minor/moderate injury, very severe injury was associated with higher IL-6 and IL-8 levels, a sign of inflammation, up to 48 h after trauma (Figure S1C), which is consistent with previously published data [11].

### 2.2. Long-Term Deficiency of ω-3 PUFAs in Traumatized Patients Compared to Controls

**Figure 1:**
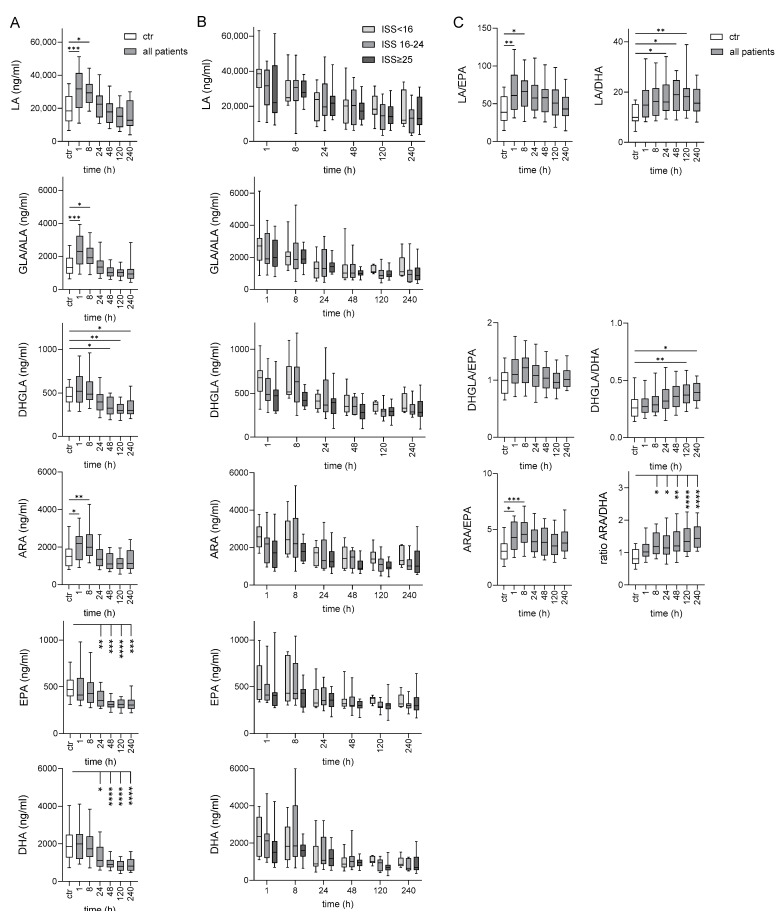
Post-traumatic time course of circulating PUFAs. (**A**) Comparison of the PUFA levels between controls and all patients (one-way ANOVA). (**B**) Comparison of the PUFA levels between patient groups (mixed effect model). (**C**) Ratio of the concentration of ω6 PUFA/ω PUFA in controls and all patients (one-way ANOVA). * *p* < 0.05, ** *p* < 0.01, *** *p* < 0.001, **** *p* < 0.0001.

Seven PUFAs were quantified in all patients and controls (Figure 1): ω-6 PUFAs LA, GLA, DHGLA, and ARA, and ω-3 PUFAs ALA, EPA, and DHA, with GLA and ALA being detected as sum parameter. There was an obvious heterogeneity in the absolute PUFA levels between the controls but also between the patients immediately after injury, likely reflecting the different (pre-injury) nutritional status. Such a variability was also observed for several PUFAs before surgical intervention [12].

Immediately, 1–8 h after injury levels of ω-6 PUFAs LA and ARA, and also of sum GLA/ALA were higher in traumatized patients compared to controls (Figure 1A). In the further posttraumatic course levels of each PUFA decreased until 120–240 h in the majority of patients. Patients showed an obvious long-term decrease for ω-3 PUFAs EPA and DHA 24–240 h after injury. There were no significant differences in any PUFA between the patient groups (Figure 1B).

Because ω-3 PUFA levels were inversely correlated with mortality risk [13], we also calculated the ratio of ω-6 LA and ARA to ω-3 EPA and DHA levels (Figure 1C). These ratios increased in the post-traumatic course, best seen for ARA/DHA. The ratios were not different between the patient groups and also similar between the various time points 1–240 h after injury.

### 2.3. Many Oxylipins, Present Transiently Immediately After Injury in Some Patients, Were Much Less Detectable or Not Detected at All in Controls

**Figure 2:**
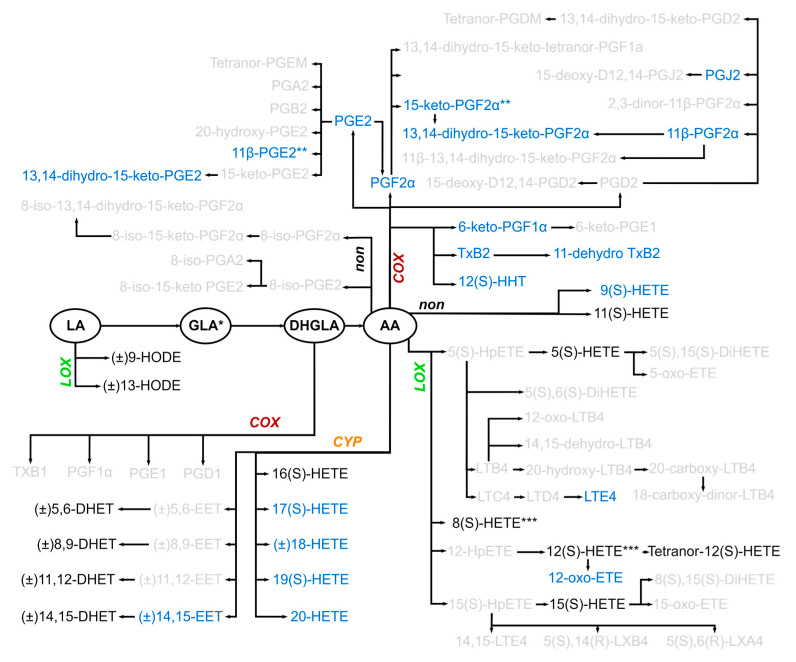
PUFA-derived oxylipins detectable in injured patients PUFAs are encircled. The metabolites are arranged mainly according to the pathway in which they are generated (cyclooxygenases, COX; lipoxygenases, LOX; cytochrome P450 mixed function oxidase enzymes, CYP; nonenzymatic free-radical mechanisms, non). Metabolites in black: present in almost all patients, metabolites in blue: present in some patients; metabolites in grey: not detected at any time point in patients. *, **, *** these PUFAs/metabolites were quantified together.

Figure S2 provides a simplified scheme of the oxylipins derived from ω-6 PUFAs that are theoretically accessible in our targeted analyses. Of the 80 targeted oxylipins 11b-PGE2 and 15-keto-PGF2a as well as 8(S)-HETE and 12(S)-HETE are detected as sum parameters. Figure 2 visualizes the oxylipins effectively detected. The main pathways involved and the relationships between PUFAs and metabolites are indicated but not all could be considered.

Around 60% of the targeted circulating oxylipins were under the detection limit in any patient and control (Table S2). A total of 13 out of 80 (13/80) of the targeted metabolites were found in almost all patients at each time point and in almost all controls. For comparisons of the oxylipin levels between controls and all patients and between patient groups, only metabolites with at least 90% detectable values of the patients in each group were considered, undetectable values were imputed. The data of these oxylipins are shown as boxplots in our study.

**Figure 3:**
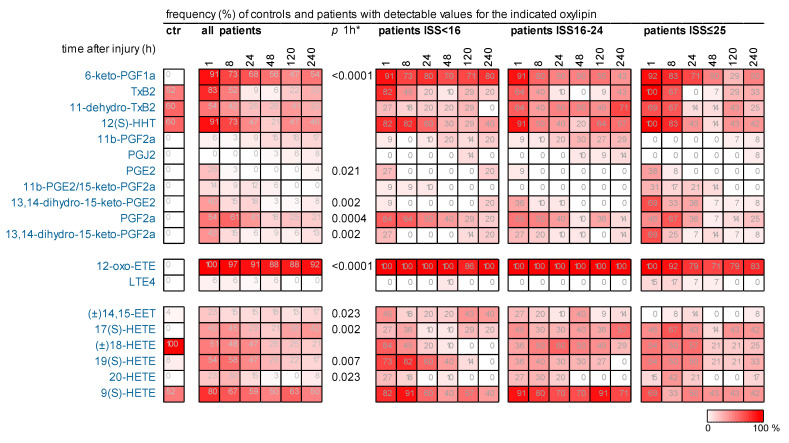
Comparison of the frequencies of controls and patients for those oxylipins that were detected in some (less than 90%) of the patients at any time point. At each time point after trauma the frequency (%) of controls (ctr), all patients, and of the 3 patient groups are indicated. * Only significant differences between controls and all patients at 1 h after injury are noted (Fisher’s exact test, *p*-value). Oxylipins present at each time point in almost all patients are not included in this figure.

20/80 of the targeted metabolites were detected in less than 90% of the patients at any time point. These oxylipins were less often (6/20) or even not detected (14/20) in controls (Table 2). For each of these oxylipins, we calculated the frequency of patients showing detectable values in the posttraumatic course and visualized these data (Figure 3). Obviously, the frequencies were, in most cases, highest immediately after injury, afterwards they declined. Thus, 1 h after injury for many of these oxylipins the frequency of patients with detectable oxylipins values was significantly higher compared to that of the controls. Figure 3 shows the data also for each patient group. However, the number of patients was too low for statistical group comparisons.

Original data of the patients and controls are provided in Table S3.

### 2.4. Late Decrease of LA-Derived 9- and 13-HODE After Very Severe Injury

In the following, we discuss the metabolites one by one depending on the PUFA from which they derive and on the main pathway in which they emerge.

**Figure 4:**
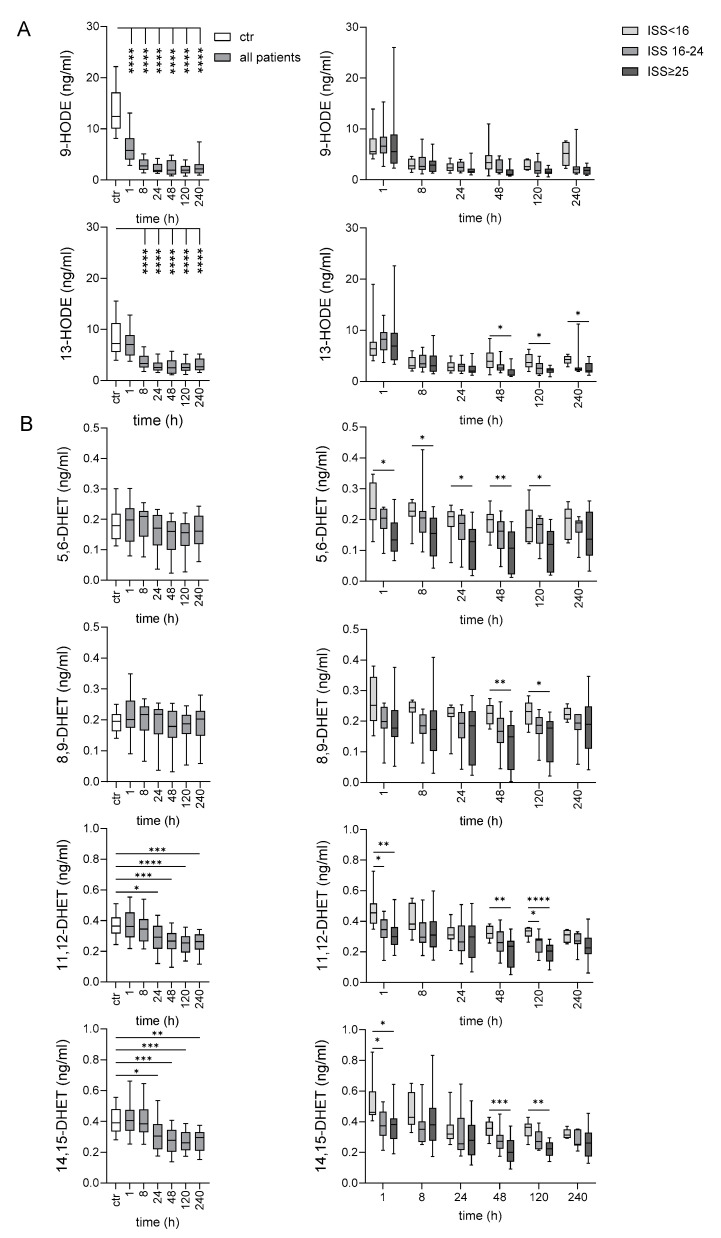
Post-traumatic time course of circulating LA-derived 9- and 13-HODE (**A**) and ARA-derived DHETs (**B**). Comparison between controls and all patients: one-way ANOVA; comparison between the indicated patient groups: mixed effect model; * *p* < 0.05, ** *p* < 0.01, *** *p* < 0.001, **** *p* < 0.0001.

Two stable monohydroxy oxidation products derived from LA, 9-hydroxyoctadecadienoic acid (HODE) and 13-HODE, were targeted. Both were quantifiable in all patients at each time point and in all controls. Compared with controls, traumatized patients showed an obvious decrease for both metabolites in the posttraumatic course from 8 h onward. Notably, 13-HODE was lower 48 h up to 240 h after trauma in patients who had sustained very severe compared to those with minor/moderate injury (Figure 4A).

### 2.5. Several Oxylipins of the COX-Generated Prostaglandin and Thromboxane Families Appeared Immediately After Injury

ARA-derived oxylipins generated in the COX pathway, which include many prostaglandins and thromboxanes, were detected in some, but not all patients and often not in controls (Figure 2 and Figure 3). 6-keto-PGF_1α_, PGE_2_, 13,14-dihydro-15-keto-PGE2, PGF_2α_, and 13,14-dihydro-15-keto-PGF_2α_ were significantly more often detected in patients 1 h after injury compared to the controls (Figure 3). When examining the patient groups, in tendency 11-dehydro-TxB_2_, 11b-PGE2/15-keto-PGF_2α_, 13,14-dihydro-15-keto-PGE_2_, and 13,14-dihydro-15-keto-PGF_2α_ were detected more often in very severe compared to less injured patients 1–8 h post-traumatically (Figure 3).

### 2.6. Early Increase in LOX-Generated ARA-Derived Oxylipins After Injury

ARA, metabolized by 5-LOX, leads to the generation of inflammatory leukotrienes including leukotriene E_4_ (LTE_4_) which was rarely detected in patients but not at all in controls (Figure 3). In this pathway, 5(S)-hydroxyeicosatetraenoic acid (HETE) was quantifiable in all patients. This was also the case for 8(S)-/12(S)-, and 15(S)-HETE, that originate from 8-, 12-, and 15-LOX activity, and for tetranor-12(S)-HETE, that derived non-enzymatically from 12(S)-HETE. These oxylipins increased immediately 1–8 h after injury in patients compared to controls. The patient groups did not differ with respect to these metabolites (Figure S3).

### 2.7. Oxylipins Generated by CYP Activity: Lower Levels of Several DHETs After Very Severe Injury

The CYP system, which utilizes ARA as a substrate, comprises two main branches of enzymes: epoxygenases and ω-hydroxylases. Members of the CYP2C and CYP2J families of CYP epoxygenases metabolize ARA to four biologically active epoxyeicosatrienoic acids (EETs): 5,6-EET, 8,9-EET, 11,12-EET, and 14,15-EET. EETs are rapidly hydrolyzed by soluble epoxide hydrolase (sEH) to more stable but less biologically active DHETs, differing in the position of the two hydroxy groups at the 20 carbon backbone (Table 2). Thus, evaluating plasma DHETs reflects the EET levels in an indirect way. 1/4 EETs, 14,15-EET, was detectable in 5/11 minor/moderately injured patients and only in 1/14 very severely injured patients 1 h after injury. The resulting four DHETs, detectable in all patients and controls, were lower in very severely compared with the minor/moderately and partly with the severely injured patients. This was not only seen immediately, 1 and partly also 8 h, but also later, 48–120 h after injury (Figure 4B). Compared to controls, traumatized patients showed an obvious decrease for 11,12-DHET and 14,15-DHET 24–240 h after injury.

The CYP4A and CYP4F ω-hydroxylase subfamilies convert ARA to 16(S)-, 17(S)-, 18-, 19(S)-, and 20-HETE (Figure 2), which were detected either in all (16(S)-HETE, Figure S3) or in some patients, respectively. In the control group 17(S)-HETE and 20-HETE were not detected, and 19(S)-HETE was less frequently present compared to all patients (Figure 3, Table 2). 18-HETE was the only metabolite detected in all controls but in less than 80% of the patients.

### 2.8. 11(S)-HETE Increased Immediately After Injury

11(S)-HETE, generated by lipid peroxidation from ARA, was detected in all patients and controls. It transiently increased immediately after injury compared to controls and decreased continuously in the further course. Very severely injured patients showed lower 11(S)-HETE levels compared to less injured patients 48–120 h after injury (Figure S3).

### 2.9. Several Oxylipins Correlate to Clinical Parameters in the Posttraumatic Course

**Figure 5:**
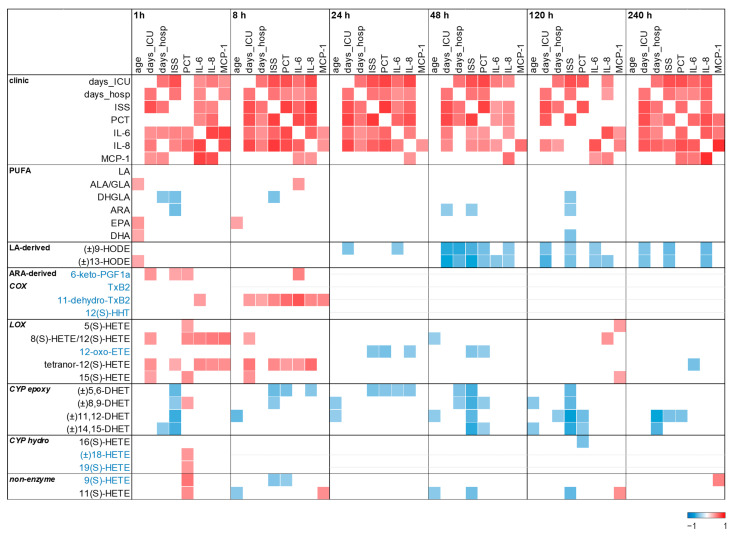
Correlation of patient’s clinical parameters with PUFA and oxylipin levels 1 to 240 h after trauma. In the correlation matrix, the square color indicates the magnitude of correlation (Spearman). Only oxylipins with significant correlations are shown. Oxylipins with less than 50% of the patients with detected values at the indicated time point were not analyzed (grey lines in the respective fields). In this analysis, values not detected in a present sample were not imputed.

Finally, we related the PUFA and oxylipin data with the clinical parameters available at each time point and visualized the significant results in a heatmap (Figure 5). As expected, PUFA levels were not informative, but the levels of several oxylipins were.

Two metabolite groups, detected in almost all patients, showed inverse relationships with injury severity, prolonged ICU and/or hospital stay, as well as PCT and/or pro-inflammatory cytokine levels, especially late in the post-traumatic course. These were LA-derived 9- and 13-HODE, which are likely to be of interest as potential biomarkers at 48 h but also up to 240 h after trauma. The second metabolite group is the various ARA-derived DHETs showing an inverse relation to the ISS and partly to other adverse clinical parameters at 1, 48, and 120 h after trauma.

Furthermore, several oxylipins arising or increasing transiently immediately after injury are directly related to adverse clinical parameters early in the post-traumatic time course; later time points could often not be examined because of the low number of patients with detectable values. Among these are ARA-derived 11-dehydroxy-TxB_2_ and tetranor-12(S)-HETE correlating to the ISS, length of stay at the ICU and/or in the hospital, and pro-inflammatory cytokine levels 1 and/or 8 h after injury.

## 3. Discussion

In the present study, we targeted postinjury 80 circulating oxylipins that partly exhibit context- and cell-type-specific pro- or anti-inflammatory properties. 16% were detected in almost all patients, while 25% could be quantified in some. Mostly, the more stable but often biologically inactive intermediates could be quantified, confirming previous studies [14].

A number of the oxylipins detected in almost all patients and controls are potential markers for the time course and outcome of traumatized patients. This concerns the first 9- and 13-HODE; their levels correlate inversely to days at ICU, ISS, and IL-6 levels late, until ten days after injury. Thus, both may predict adverse outcomes after trauma. HODEs are generated by LOXs or non-enzymatically, and their pro- or anti-inflammatory biological effects are context-specific [15]. Exemplarily, in early atherosclerosis, 13-HODE is generated in macrophages enzymatically by 15-LOX-1. This probably activates protective mechanisms that increase the clearance of lipid and cellular debris from the vessel wall. In later atherosclerosis, non-enzymatic oxidation of LA generates both 9- and 13-HODE. At this stage of the disease, and with the pro-inflammatory actions of 9-HODE after binding to GPR132, the effects of HODEs may be predominantly harmful.

Secondly, DHETs, the stable inactive derivatives of the bioactive EET regioisomers may be such markers. Patients with the most severe injuries exhibited the lowest levels of the resulting DHETs up to 120 h after injury. Traumatized patients were deficient in 11,12- and 13,14-DHET late after injury when compared with controls. EETs, identified as autocrine and paracrine signaling lipid mediators, regulate diverse biological processes (reviewed in [16]). Notably, they mediate a number of anti-inflammatory effects [16,17,18,19]. Exemplarily, 11,12 EET rescued deteriorated wound healing in a combined model of hyperglycemia and ischemia by resolution of inflammation and augmentation of neovascularization [20]. Because EETs are highly unstable and rapidly converted by sEHs to DHET, approaches to increase EET levels were conducted [16,18,19,20,21,22,23]. Experimentally, the potentiation of EET activity can be achieved in various ways in mice, including loss of sEH or endothelial-specific overexpression of human *CYPJ2* or *CYP2C8*. This inhibited inflammatory gene expression and signaling pathways in endothelial cells and monocytes, as well as in models of cardiovascular diseases. The sEH inhibitor 1-(1-propanoylpiperidin-4-yl)-3-[4-(trifluoromethoxy)phenyl]urea (TPPU) dampened inflammation and the generation of DHET in a burned mouse model [21]. In human studies, the application of stable inhibitors of sEH has been employed to enhance the availability of circulating EETs in patients [23].

Several metabolites immediately present after injury in a part of the traumatized patients were not detected in any control. These frequently injury-dependent occurring oxylipins belong to ARA-derived metabolites mainly generated by COX-, partly also by LOX-activity, with primarily pro-inflammatory properties such as PGE_2_ and its derivative 13,14-dihydro-15-keto-PGE_2_. Immediately after trauma oxylipins with pro-inflammatory properties tended to be more often detectable in patients with an ISS ≥ 25 compared to patients with a lower ISS. 13,14-dihydroxy-15keto-PGE_2_, 13,14-dihydroxy-15keto-PGF_2α_ and 11β-PGE2/15-keto-PGF_2α_ are widely considered as stable, inactive forms of PGE_2_ or PGF_2α_, but recent data show that 15-keto-PGE_2_ is bioactive and could terminate PGE_2_–evoked signaling [24,25]. Furthermore,11-dehydro-TxB_2_ was more frequently detected immediately after severe and very severe injury. It is produced from the breakdown of TxB_2_, a stable, inactive degradation product of TxA_2_. TxA_2_ is synthesized by platelets in response to native or exogenous added stimuli. In the past, PGF_2α_, PGE_2_, and/or TxB_2_ have already been associated with trauma, either in experimental models or patients. In rat spinal cord injury models, TxB_2_ increased in the spinal cord [26], and PGE_2_ and TxB_2_ were elevated in cerebrospinal fluid [27]. In humans, TxB_2_ was found to be elevated at admission to the hospital after traumatic brain injury [28] and in patients with recent major trauma or active sepsis [29].

Notably, around 60% of the targeted circulating metabolites were under the detection limit in any patient and control in our study. This concerns many unstable intermediates of COX- or non-enzymatically generated prostaglandins or of LOX-generated leukotrienes and lipoxins. Nearly all of these oxylipins were also not detected, if targeted, in other studies quantifying oxylipins in human plasma [30].

Initially, studies investigating oxylipins after trauma focused on brain injury because the brain is particularly rich in long-chain PUFAs, especially in excitable membranes [31]. Experimental models permit the direct quantification of oxylipin release in the brain [32,33]. In a pediatric rat model, within the first hour following trauma, brain oxylipins surge with numerous eicosanoids and octadecanoids [32]. Afterward, their levels decrease, although some remain elevated. A similar posttraumatic time course was seen for many circulating oxylipins in our study. Furthermore, the use of 3D MALDI-MSI to visualize lipid distribution in sectioned injured rat brains demonstrated lipid changes associated with traumatic brain injury and identified lesion-specific lipids [33]. In humans, after traumatic brain injury, the levels of ARA, DHA, 5-HETE, and 12-HETE, the only PUFAs/oxylipins targeted in the cited study, were 10-fold higher in the cerebral spinal fluids of patients compared to controls [34].

Profiling circulating oxylipids is challenging. To address the low abundance and instability of released oxylipins in blood, Orr et al. [35] evaluated the transcriptome of circulating leukocytes in trauma patients. The expression of 18 genes critical to the synthesis, signaling, and metabolism of pro-inflammatory oxylipins and SPMs was quantified in 96 patients after blunt trauma with uncomplicated and complicated recoveries. Patients with complicated courses and worse clinical outcomes exhibited higher expression ratios of leukotriene to resolvin pathway genes. However, transcriptomic profiling cannot reflect the high diversity of released oxylipids as detected in our patients.

In summary, the PUFA-derived oxylipidome of injured patients differs obviously from that of uninjured controls. The posttraumatic course is characterized by an early enhanced release of pro-inflammatory ARA-derived, COX-and LOX-generated oxylipins, followed by a subsequent decrease in 9- and 13-HODE, as well as in 11,12- and 13,14-DHET. The levels of several oxylipins correlate to clinical parameters.

### Limitation of the Study

Our study focused on ω-6 derived metabolites. ω-3 EPA and DHA can be metabolized to LXA_5_ and E-resolvins and D-resolvins, protectins, and maresins, respectively [36,37]. These oxylipins, not having been targeted here, are characterized as specialized pro-resolving mediators (SPMs), terminating inflammation by exerting anti-inflammatory and pro-resolving activities (reviewed in [37]). However, numerous studies show that SPMs are only found at extremely low levels or are even undetectable in biological samples [38,39,40].

The levels of certain oxylipins are related to age [41,42], a fact not considered in our study. Three major 12-LOX products were present at high levels in young females (20–55 years) compared with older females and males (55+) [42]. The median age of our patients and controls was ~58 years.

Overall, the sample sizes are quite small, limiting statistical power. The number of patients, especially those with very severe injuries, was too low to verify the suitability of several detected oxylipins as potential markers for the posttraumatic course and outcome. It would be beneficial to include additional clinical parameters, such as the timely-matched white blood cell count and daily clinical SOFA, APACHE II, and SAPS3 scores over the whole post-traumatic course. This would allow a more detailed evaluation of circulating oxylipins in relation to the clinical context.

## 4. Materials and Methods

### 4.1. Clinical Study on Traumatized Patients and Controls

We included 36 injured patients in our study (Table 1 and Table S1). Exclusion criteria were age < 18 years, life expectancy < 24 h, participation in other studies, cardiopulmonary reanimation on the accident scene or death immediately after hospital admission, known or suspected pregnancy, and radiotherapy or chemotherapy within the previous 3 months. The ISS was estimated after whole-body computed tomography and verified retrospectively. By definition, an ISS of 1–8 is considered minor, 9–15 moderate, 16–24 severe, and ≥25 very severe [10]. The SOFA score, the APACHE II score, SAPS3, and GCS were calculated at 1, 8, and 24 h. In patients with an ISS < 16, daily scores could not be applied after 24 h because the necessary score parameters were not gathered.

We included a control group (n = 25) of an age- (median 60.5 (50.3–69.3) years) and sex-matched (9 females) cohort from the LIFE-Leipzig-Heart study [43,44]. The blood donors of this control group had normal values in all basic clinical parameters such as pro-inflammatory markers, fat metabolic parameters, enzyme levels, or white blood cell count, and showed angiographically unsuspicious coronary arteries.

### 4.2. Blood Sample Preparation

Blood was collected from the injured patients 1, 8, 24, 48, 120, and 240 h after hospital admission. Tolerance for blood taking was ±10% per time point. The control group had an immediate blood taking. To obtain EDTA plasma or serum, the respective monovette (Sarstedt AG, Nümbrecht, Germany) was centrifuged at 2000× *g* for 10 min at 20 °C. The supernatants were transferred to coded cryotubes, stored at −80 °C, and thawed only once because many metabolites are sensitive to inaccurate processing of blood samples. Influencing factors such as pre-centrifugation delay > 2 h, long-term storage above −80 °C, or repeated freeze–thaw cycles may lead to a significant increase in metabolites [7,9].

### 4.3. LC-MS/MS of PUFAs and Oxylipins

Targeted LC-MS/MS analysis for quantification of 7 PUFAs and 80 oxylipins was performed according to our previously published method with modifications [7,8]. Separate LC-MS/MS methods for PUFAs and for oxylipins were applied. The oxylipin method was transferred to a more sensitive mass spectrometer. Chromatographic separation was optimized for each method.

Both methods could be conducted after the same sample preparation. In brief, 100 µL plasma was mixed with 225 µL precipitation solution containing the internal standards (50 ng/mL for PUFAs and 5 ng/mL for oxylipins). After vortexing for 2 min, samples were centrifuged for 5 min at 10,000× *g*, and the clear supernatant was transferred into independent aliquots for each method. Separate seven-point calibration was performed in the concentration range of 100–10,000 ng/mL for PUFAs and 100–10,000 pg/mL for each oxylipin.

The LC system from Shimadzu (Duisburg, Germany) was similar for both methods and consisted of a Prominence UFLC system with two high-pressure gradient pumps (LC-20ADXR), an isocratic pump (LC-20AD), a column oven (CTO-20AC), a controlling module (CBM-20A), and a HTS PAL autosampler from CTC Analytics (Zwingen, Switzerland).

An on-line SPE was implemented in both methods using a Strata-X extraction column (20 × 2 mm i.d., 25 µm particle size; Phenomenex, Aschaffenburg, Germany) and MeOH/water/FA 10:90:0.02 *v*/*v*/*v*, with a flow rate of 3 mL/min for 1 min. Chromatographic separation for both methods was performed on a core-shell LC column (Kinetex C_18_, 100 × 2.1 mm i.d., 2.6 µm particle size; Phenomenex).

For PUFAs, the flow rate was set to 0.5 mL/min and the gradient was applied as follows: 0 to 100% eluent B in 8.8 min (eluent A: water/ACN/FA 63:37:0.02 *v*/*v*/*v*, eluent B: iPrOH/ACN 50:50 *v*/*v*), 100% B for 1.2 min, re-equilibration of the columns for 2 min. The column oven was kept at 35 °C. Injection volume was 10 µL. The LC system was coupled to a QTRAP^®^ 5500 mass spectrometer (SCIEX, Framingham, MA, USA) equipped with a Turbo V™ ion spray source operating in negative ion mode.

For oxylipins, the flow rate was set to 0.7 mL/min and the gradient was applied as follows: 15 to 100% eluent B in 13.1 min (eluent A: water/MeOH 90:10 *v*/*v*, eluent B: MeOH/water 90:10 *v*/*v*), 100% B for 2 min, re-equilibration of the columns for 2 min. The column oven was kept at 50 °C. Injection volume was 10 µL. The LC system was coupled to a QTRAP^®^ 6500+ mass spectrometer (SCIEX) equipped with a Turbo V™ ion spray source operating in negative ion mode.

Table 2 and Table S2 give an overview and useful information on the targeted PUFAs and oxylipins.

### 4.4. Quantitation of Further Parameters

IL-6, IL-8, and MCP-1 (CCL2) were quantified in serum using the human IL-6, IL-8, and MCP-1 BD OptEIA ELISA kits (BD Life Sciences, Heidelberg, Germany). Procalcitonin (PCT) was quantified in plasma using the TRACE technology (Roche Pharma AG, Grenzach-Wyhlen, Germany).

### 4.5. Statistical Analyses

The absolute levels of PUFAs and metabolites were provided. For metabolites not present in a measured sample, values below the limit of detection were imputed with half the minimal measured value [45]. This imputation was conducted only for metabolites with at least 10% values less than the limit of detection at the indicated time point. If no patient sample was available at a certain time point because the patient underwent surgery or already left the hospital, this sample was treated as missing. For continuous variables median and the interquartile range [25th–75th percentile] were used. Data were analyzed using SPSSv27 (IBM, Armonk, NY, USA) and GraphPad Prism10.2 (GraphPad Software, Boston, MA, USA). Box plots are shown as mean/25–75% percentiles with 10–90 percentile whiskers. *p* values were two-sided and α < 0.05 was used for hypothesis testing. We corrected for multiple comparisons by controlling the False Discovery Rate using the Two-stage step-up method of Benjamini, Krieger, and Yekutieli.

## Figures and Tables

**Table 1:** Patients’ characteristics grouped according to the ISS.

	Traumatized Patients			*p* Value *		
	Group 1, ISS < 16	Group 2, ISS 16–24	Group 3, ISS ≥ 25	Comparing Group		
patients (n)	11	11	14	1–2	2–3	1–3
ISS	12.5 (9.0–13.0)	20.0 (19.0–21.0)	41.0 (34.0–50.0)	0.043	0.010	<0.001
age (years)	55.5 (32.0–68.0)	60.0 (35.0–70.0)	53.0 (34.0–59.0)	0.849	0.599	0.455
sex, female (n)	1	3	7	1	1	0.126
days at ICU (n)	2.5 (2.0–3.0)	2.0 (2.0–8.0)	15.0 (6.0–34.0)	1	0.004	0.002
days in hospital (n)	12.0 (7.0–16.0)	14.0 (12.0–17.0)	23.0 (16.0–42.0)	0.732	0.217	0.007
death in hospital (n)	0	0	2			
SOFA score at 24 h	1.0 (1.0–3.0)	3.0 (1.0–4.0)	7.0 (5.0–9.0)	0.880	0.087	0.003

Values are given as median with interquartile range. * Kruskal–Wallis H test; Sequential Organ Failure Assessment (SOFA).

**Table 2:** Targeted PUFAs and PUFA-derived oxylipins were detected in injured patients and controls.

	PUFA/Metabolite	Present in Controls (n = 25)	PUFA/Metabolite Derived From	1st; 2nd Pathway	Fatty Acid	Molecular Formula
1	LA	25	PUFA		ω 6	C18H32O2
2	GLA/ALA *	25	PUFA		ω 6	C18H30O2
3	DHGLA	25	PUFA		ω 6	C20H34O2
4	ARA	25	PUFA		ω 6	C20H32O2
5	EPA	25	PUFA		ω 3	C20H30O2
6	DHA	25	PUFA		ω 3	C22H32O2
7	9-HODE	25	LA	LOX; peroxidation	ω 6	C18H32O3
8	13-HODE	25	LA	LOX; peroxidation	ω 6	C18H32O3
9	6-keto-PGF1a	-	ARA	COX	ω 6	C20H34O6
10	TxB2	13/25	ARA	COX2	ω 6	C20H34O6
11	11-dehydro-TxB2	15/25	ARA	COX2	ω 6	C20H32O6
12	12(S)-HHT	13/25	ARA	COX	ω 6	C17H28O3
13	11b-PGF2a	-	ARA	COX2	ω 6	C20H34O5
14	PGJ2	-	ARA	COX	ω 6	C20H32O5
15	PGE2	-	ARA	COX	ω 6	C20H32O5
16	11b-PGE2/15-keto-PGF2a **	-	ARA	COX2	ω 6	C20H32O5
17	13,14-dihydro-15-keto-PGE2	-	ARA	COX	ω 6	C20H32O5
18	PGF2a	-	ARA	COX2	ω 6	C20H34O5
19	13,14-dihydro-15-keto-PGF2a	-	ARA	COX2	ω 6	C20H34O5
20	5(S)-HETE	25	ARA	LOX; CYP	ω 6	C20H32O3
21	8(S)-HETE/12(S)-HETE ***	25	ARA	LOX; CYP	ω 6	C20H32O3
22	12-oxo-ETE	-	ARA	LOX	ω 6	C20H30O3
23	tetranor-12(S)-HETE	25	ARA	peroxidation; LOX	ω 6	C16H26O3
24	15(S)-HETE	25	ARA	15-LOX-1/2; CYP	ω 6	C20H32O3
25	LTE4	-	ARA	5-LOX	ω 6	C23H37NO5S
26	5,6-DHET	24/25	ARA	CYP	ω 6	C20H34O4
27	8,9-DHET	24/25	ARA	CYP	ω 6	C20H34O4
28	11,12-DHET	25	ARA	CYP	ω 6	C20H34O4
29	14,15-EET	1/25	ARA	CYP	ω 6	C20H32O3
30	14,15-DHET	25	ARA	CYP	ω 6	C20H34O4
31	16(S)-HETE	25	ARA	CYP	ω 6	C20H32O3
32	17(S)-HETE	-	ARA	CYP	ω 6	C20H32O3
33	18-HETE	25	ARA	CYP/LOX	ω 6	C20H32O3
34	19(S)-HETE	2/25	ARA	CYP	ω 6	C20H32O3
35	20-HETE	-	ARA	CYP	ω 6	C20H32O3
36	9(S)-HETE	13/25	ARA	peroxidation; CYP	ω 6	C20H32O3
37	11(S)-HETE	25	ARA	peroxidation; COX, LOX	ω 6	C20H32O3

PUFA and metabolites detected in almost all patients are in black, and those detected in some patients are in blue; *, **, *** these PUFAs/metabolites were quantified together.

## Data Availability

The general patient data and quantitative data of the analyzed clinical and LC-MS/MS parameters are provided in the Supplementary Materials, Table S3.

## Supplementary Materials

The following supporting information can be downloaded at: https://www.mdpi.com/article/10.3390/ijms252413530/s1.

## Abbreviations

Acute Physiology And Chronic Health Evaluation (APACHE), base excess concentration of extracellular fluid (BE), Glasgow Coma Scale (GCS), injury severity score (ISS), intensive care unit (ICU), pattern-recognition receptor (PRR), phospholipase A (PLA), polyunsaturated fatty acids (PUFA), procalcitonin (PCT), soluble epoxide hydrolase (sEH), Simplified Acute Physiology Score 3 (SAPS3), Sequential Organ Failure Assessment (SOFA), and specialized pro-resolving mediator (SPM). PUFAs and Oxylipins: alpha-linolenic acid (ALA), arachidonic acid (ARA), cyclooxygenase (COX), cytochrome P450 mixed function oxidase (CYP), docosahexaenoic acid (DHA), dihydroxyeicosatrienoic acid (DHET), dihomo-gamma-linolenic acid (DHGLA), epoxyeicosatrienoic acid (EET), eicosapentaenoic acid (EPA), eicosatetraenoic acid (ETE), gamma-linolenic acid (GLA), hydroxyeicosatetraenoic acid (HETE), hydroxyheptadecatrienoic (HHT), hydroxyoctadecadienoic acid (HODE), linoleic acid (LA), lipoxygenase (LOX), leukotriene (LT), lipoxin (LX), prostaglandin (PG), phospholipase A (PLA), soluble epoxide hydrolase (sEH), and thromboxane (Tx).
