# Supplementary material for: Severity-Dependent Long-Term Post-Traumatic Changes in the Circulating Oxylipin Profile
Source: Int J Mol Sci. 2024 Dec 17;25(24):13530. doi: 10.3390/ijms252413530 (PMC11680030; doi:10.3390/ijms252413530)
Supplement: Supplementary file 1 [file ijms-25-13530-s001.zip › ijms-3380796-supplementary.pdf]

Supplementary Information

Supplementary figure 1 (Fig. S1) Characteristics of the injured patients

Supplementary figure 2 (Fig. S2) Schemes of the targeted PUFA-derived oxylipodome pathways

Supplementary figure 3 (Fig. S3) Posttraumatic time course of circulating ARA-derived oxylipins mainly generated by LOX activity

Supplementary table S1 (Tab. S1) Trauma classification based on the abbreviated injury scale (AIS)

Supplementary table S2 (Tab. S2) Targeted PUFA-derived oxylipins not detected in injured patients

Supplementary table S3 (Tab. S3) Original data of trauma patients and controls

Supplementary figures

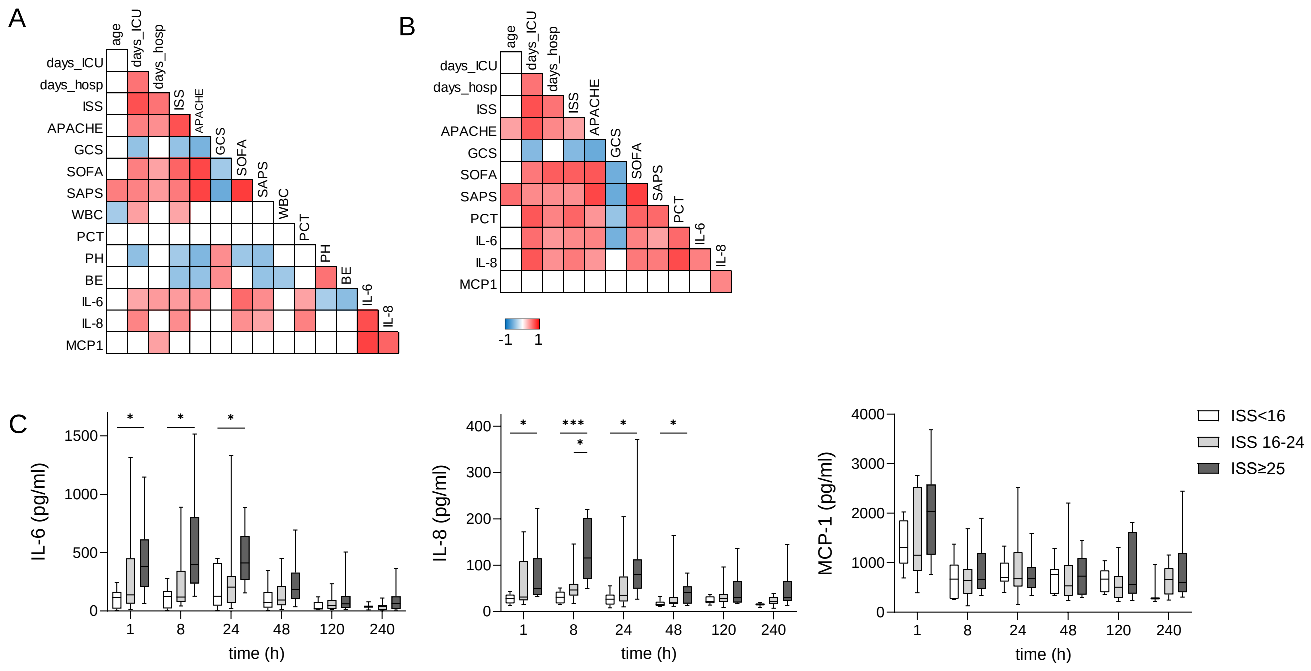

Fig. S1 Characteristics of the analyzed injured patients

A, **B** Correlation of patient's clinicopathological parameters 1 h (**A**) and 24 h (**B**) after trauma. In the correlation matrix square color indicates the magnitude of correlation (Spearman). Only parameters which are exactly time-matched to blood taking for PUFA/metabolite analyses and only significant correlations are shown; Acute Physiology and Chronic Health Evaluation II (APACHE II) score, base excess concentration of extracellular fluid (BE), Glasgow Coma Scale (GCS), intensive care unit (ICU), precalcitonin (PCT), Simplified Acute Physiology Score 3 (SAPS3), Sequential Organ Failure Assessment (SOFA) score, white blood cell count (WBC).

C Posttraumatic time course of circulating cytokines. Comparison between patient groups: mixed effect model, *p<0.05; ***p<0.001.

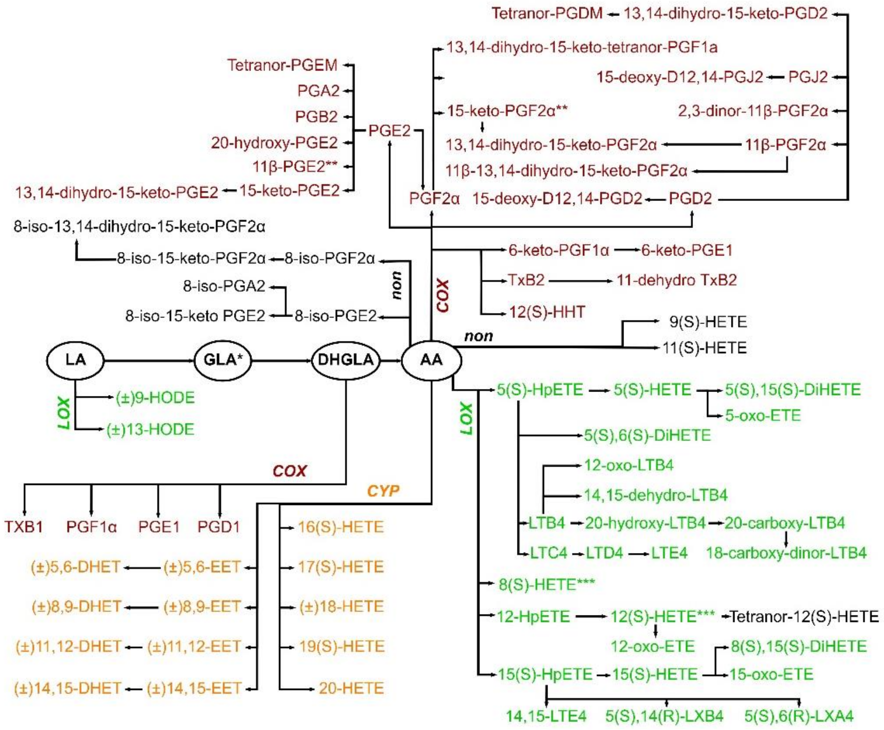

Fig. S2 Schemes of the targeted PUFA-derived oxylipodome pathways

PUFAs are encircled. Three main enzymatic pathways are involved in the release of the PUFA-derived metabolites: cyclooxygenases (COX, red), lipoxygenases (LOX, green); and cytochrome P450 mixed function oxidase enzymes (CYP, orange). Metabolites mainly derived from nonenzymatic free-radical (non) mechanisms are in black. The authors are aware that the scheme does not show all possible pathways and pathway steps. For further explanations see „Introduction".

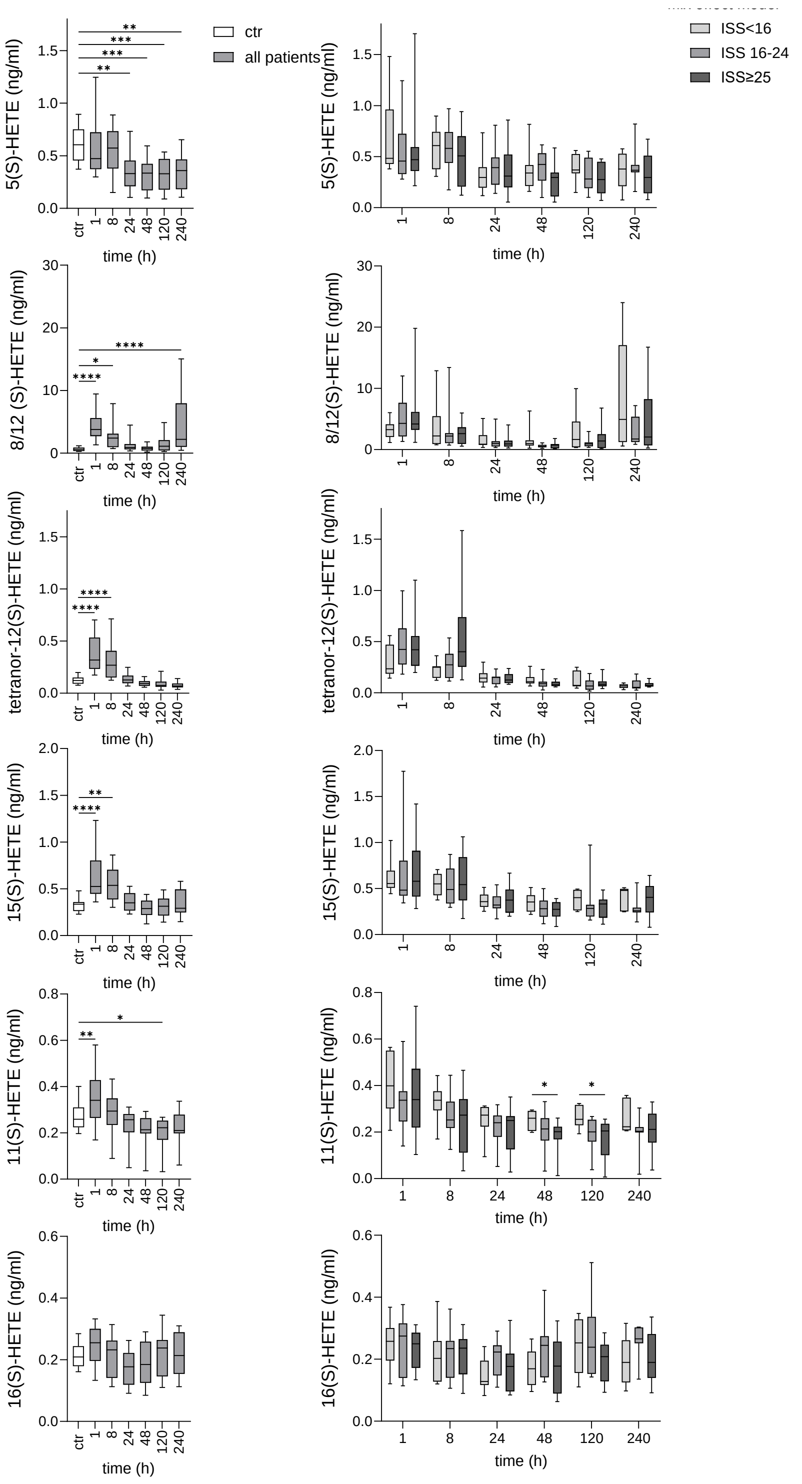

Fig. S3 Posttraumatic time course of circulating ARA-derived oxylipins mainly generated by LOX activity.
Comparison between controls and all patients: one-way ANOVA, comparison between the indicated patient groups: mixed effect model; *p<0.05, **p<0.01, ***p<0.001, ****p<0.000.1.

Supplementary tables

Tab. S1 Trauma classification based on the abbreviated injury scale (AIS)

Trauma group	Injuries	group 1 (ISS <16)	group 2 (ISS 16-24)	group 3 (ISS ≥25)	p-value*	
					groups 1-3	groups 2-3
Neurotrauma	traumatic brain injury	3	3	7	0.414	0.414
	spinal cord injury	0	0	3	0.230	0.230
Facial trauma	eye injury	0	2	1	1	0.565
	fractured face	1	3	5	0.180	1
	open wound face	0	0	1	1	1
Neck trauma	esophageal injury	0	0	0	1	1
	laryngeal trauma	0	1	0	1	0.440
	tracheal trauma	0	0	0	1	1
Thoracic trauma	internal organs injury	2	3	11	0.005	0.017
	other injuries	5	2	12	0.081	0.001
Abdominal trauma	internal organs injury	0	1	9	0.001	0.012
	other injuries	0	0	1	1	1
Spinal trauma	fracture	2	2	10	0.015	0.015
	luxation/distortion	0	0	2	0.487	0.487
Pelvic trauma	fracture	1	5	7	0.042	1
Upper extremities trauma	fracture	4	4	8	0.428	0.428
	luxation/distortion	1	4	2	1	0.350
Lower extremities trauma	fracture	4	4	9	0.238	0.238
	luxation/distortion	1	6	2	1	0.081

Fisher's exact test; groups 1 and 2 were not different

Tab. S2 Targeted PUFA-derived oxylipins not detected in injured patients or controls

	**metabolite**	**derived from**	**main pathway**	**molecular formula**
1	TxB1	DHGLA	COX	C20H36O6
2	PGD1	DHGLA	COX	C20H34O5
3	PGE1	DHGLA	COX	C20H34O5
4	PGF1a	DHGLA	COX	C20H36O5
5	6-keto-PGE1	ARA	COX	C20H32O6
6	PGD2	ARA	COX	C20H32O5
7	15-deoxy-D12,14-PGD2	ARA	COX	C20H30O4
8	13,14-dihydro-15-keto-PGD2	ARA	COX	C20H32O5
9	tetranor-PGDM	ARA	COX	C16H24O7
10	11b-13,14-dihydro-15-keto-PGF2a	ARA	COX	C20H34O5
11	2,3-dinor-11β-PGF2α	ARA	COX	C18H30O5
12	15-deoxy-D12,14-PGJ2	ARA	COX	C20H28O3
13	tetranor-PGEM	ARA	COX	C16H24O7
14	PGA2	ARA	COX	C20H30O4
15	PGB2	ARA	COX	C20H30O4
16	20-hydroxy-PGE2	ARA	COX	C20H32O6
17	15-keto-PGE2	ARA	COX	C20H30O5
18	20-hydroxy-PGF2a	ARA	COX	C20H34O6
19	13,14-dihydro-15-keto PGF1α	ARA	COX	C20H36O5
20	(±)5,6-EET	ARA	CYP	C20H32O3
21	(±)8,9-EET	ARA	CYP	C20H32O3
22	(±)11,12-EET	ARA	CYP	C20H32O3
23	5(S)-HpETE	ARA	LOX	C20H32O4
24	5-oxo-ETE	ARA	LOX	C20H30O3
25	5(S),6(S)-DiHETE	ARA	LOX	C20H32O4
26	5(S),15(S)-DiHETE	ARA	LOX	C20H32O4
27	LTB4	ARA	LOX	C20H32O4
28	12-oxo-LTB4	ARA	LOX	C20H30O4
29	14,15-dehydro-LTB4	ARA	LOX	C20H30O4
30	20-hydroxy-LTB4	ARA	LOX	C20H32O5
31	20-carboxy-LTB4	ARA	LOX	C20H30O6
32	18-carboxy-dinor-LTB4	ARA	LOX	C18H26O6
33	LTC4	ARA	LOX	C30H47N3O9S
34	LTD4	ARA	LOX	C25H40N2O6S
35	12(S)-HpETE	ARA	LOX	C20H32O4
36	15(S)-HpETE	ARA	LOX	C20H32O4
37	8(S),15(S)-DiHETE	ARA	LOX	C20H32O4
38	15-oxo-ETE	ARA	LOX	C20H30O3
39	5(S),6(R)-LXA4	ARA	LOX	C20H32O5
40	5(S),14(R)-LXB4	ARA	LOX	C20H32O5
41	14,15-LTE4	ARA	LOX	C23H37NO5S
42	8-iso-PGA2	ARA	peroxidation	C20H30O4
43	8-iso-PGE2	ARA	peroxidation	C20H32O5
44	8-iso-15-keto-PGE2	ARA	peroxidation	C20H30O5
45	8-iso-PGF2a	ARA	peroxidation	C20H34O5
46	8-iso-15-keto-PGF2a	ARA	peroxidation	C20H32O5
47	8-iso-13,14-dihydro-15-keto-PGF2a	ARA	peroxidation	C20H34O5

Tab. S3 Original data of trauma patients and controls

Patients characteristics									clinical lab parameters				poly unsaturated fatty acids (PUFAs)						detected oxylipins																														
Trauma patient / control	Patient ID	Time point	Age [years]	Gender	days at ICU	days in hospital	ISS	ISS group	PCT [ng/mL]	IL-6 [pg/mL]	IL-8 [pg/mL]	MCP-1 [pg/mL]	LA [ng/mL]	ALA/GLA [ng/mL]	DHGLA [ng/mL]	ARA [ng/mL]	EPA [ng/mL]	DHA [ng/mL]	(±)9-HODE [ng/mL]	(±)13-HODE [ng/mL]	(±)5,6-DHET [ng/mL]	(±)8,9-DHET [ng/mL]	(±)11,12-DHET [ng/mL]	(±)14,15-EET [ng/mL]	(±)14,15-DHET [ng/mL]	16(S)-HETE [ng/mL]	17(S)-HETE [ng/mL]	(±)18-HETE [ng/mL]	19(S)-HETE [ng/mL]	20-HETE [ng/mL]	6-keto-PGF1α [ng/mL]	TxB2 [ng/mL]	11-dehydro-TxB2 [ng/mL]	12(S)-HHT [ng/mL]	11β-PGF2α [ng/mL]	PGJ2 [ng/mL]	PGE2 [ng/mL]	11β-PGE2/ 15-keto-PGF2α [ng/mL]	13,14-dihydro- 15-keto-PGE2 [ng/mL]	PGF2α [ng/mL]	13,14-dihydro- 15-keto-PGF2α [ng/mL]	5(S)-HETE [ng/mL]	8(S)-HETE/ 12(S)-HETE [ng/mL]	12-oxo-ETE [ng/mL]	Tetranor- 12(S)-HETE [ng/mL]	15(S)-HETE [ng/mL]	LTE4 [ng/mL]	9(S)-HETE [ng/mL]	11(S)-HETE [ng/mL]
Trauma patient	1	1	55	male	3	14	9	1	0.02	24	35	1898	30533	2287	403	1861	360	1198	12.3	7.74	0.15	0.19	0.41	<LOD	0.41	0.27	<LOD	<LOD	<LOD	<LOD	0.22	<LOD	<LOD	<LOD	<LOD	<LOD	<LOD	<LOD	0.08	0.09	0.12	0.48	4.88	5.09	0.14	1.06	<LOD	<LOD	0.26
Trauma patient	6	1	73	female	2	16	10	1	0.02	-777	-777	-777	66248	6754	1062	3912	1015	3849	14.3	21.7	0.22	0.29	0.52	<LOD	0.52	0.29	0.16	0.11	0.90	<LOD	0.01	0.32	<LOD	1.20	<LOD	<LOD	0.03	<LOD	<LOD	<LOD	0.14	1.21	1.06	0.38	0.15	0.59	<LOD	<LOD	0.40
Trauma patient	11	1	30	male	3	10	14	1	0.07	147	44	1840	41327	2705	762	2599	469	2235	6.99	7.10	0.35	0.39	0.61	<LOD	0.77	0.26	<LOD	0.12	2.36	2.28	0.05	0.13	<LOD	0.66	<LOD	<LOD	<LOD	<LOD	<LOD	0.15	<LOD	1.55	3.27	3.25	0.49	0.89	<LOD	0.46	0.55
Trauma patient	12	1	32	male	3	17	13	1	0.04	36	23	921	50732	1813	692	3128	352	1440	5.52	5.06	0.26	0.25	0.38	0.18	0.41	0.10	<LOD	<LOD	0.70	1.44	0.15	1.42	<LOD	3.82	<LOD	<LOD	0.26	0.02	<LOD	0.09	0.10	0.37	4.11	3.44	0.21	0.55	<LOD	0.28	0.57
Trauma patient	13	1	69	male	5	16	13	1	0.04	121	33	2038	32817	2980	530	2320	728	3293	5.37	8.12	0.24	0.25	0.39	<LOD	0.44	0.20	<LOD	<LOD	<LOD	<LOD	0.19	<LOD	<LOD	0.01	<LOD	<LOD	<LOD	<LOD	<LOD	<LOD	<LOD	0.41	6.32	1.39	0.58	0.52	<LOD	0.32	0.30
Trauma patient	17	1	26	male	2	6	13	1	0.02	11	12	1220	40655	2513	677	2028	334	1276	8.12	7.65	0.25	0.28	0.50	0.16	0.55	0.21	<LOD	<LOD	1.17	<LOD	0.14	0.74	<LOD	2.58	<LOD	<LOD	<LOD	<LOD	<LOD	0.09	<LOD	0.45	2.73	0.60	0.19	0.51	<LOD	0.33	0.43
Trauma patient	18	1	38	male	0	6	6	1	0.04	6.0	17	665	38631	2764	652	2577	438	1088	5.22	5.44	0.32	0.35	0.76	<LOD	0.87	0.34	<LOD	0.12	2.31	<LOD	0.13	0.26	<LOD	0.98	<LOD	<LOD	<LOD	<LOD	<LOD	0.08	<LOD	0.79	1.55	0.90	0.23	0.46	<LOD	0.38	0.42
Trauma patient	19	1	56	male	3	7	14	1	0.08	205	36	1395	37446	3600	733	3202	696	3408	6.85	6.37	0.33	0.35	0.48	0.17	0.60	0.19	<LOD	0.05	1.08	<LOD	0.17	0.88	<LOD	2.74	<LOD	<LOD	0.07	<LOD	<LOD	0.11	<LOD	0.96	4.15	1.32	0.33	0.70	<LOD	0.37	0.56
Trauma patient	25	1	34	male	2	7	12	1	0.07	151	18	1000	18207	1098	520	2205	380	2353	4.56	3.87	0.20	0.20	0.44	0.17	0.46	0.30	0.13	0.08	0.80	<LOD	0.34	1.00	0.11	2.47	<LOD	<LOD	<LOD	<LOD	<LOD	<LOD	<LOD	0.47	3.78	0.30	0.24	0.44	<LOD	0.31	0.33
Trauma patient	29	1	68	male	2	37	8	1	0.02	248	18	1606	9547	804	297	1662	593	2382	3.96	4.90	0.21	0.22	0.46	0.18	0.46	0.37	0.14	0.06	<LOD	<LOD	0.16	1.03	0.09	2.54	0.18	<LOD	<LOD	<LOD	<LOD	<LOD	<LOD	0.55	3.25	0.70	0.28	0.53	<LOD	0.29	0.37
Trauma patient	36	1	64	male	2	3	5	1	0.06	109	39	1108	38529	3203	953	3077	917	3995	5.02	6.21	0.12	0.14	0.34	<LOD	0.46	0.21	<LOD	<LOD	0.50	0.65	0.09	0.32	0.06	0.92	<LOD	<LOD	<LOD	<LOD	<LOD	0.06	<LOD	0.43	2.06	0.23	0.47	0.61	<LOD	0.19	0.19
Trauma patient	7	1	35	female	2	14	21	2	0.02	93	23	409	10704	856	280	1174	326	927	2.43	3.45	0.11	0.19	0.30	<LOD	0.31	0.25	<LOD	<LOD	<LOD	<LOD	0.26	0.67	<LOD	2.30	<LOD	<LOD	<LOD	<LOD	0.08	0.13	0.09	0.37	4.30	1.39	0.31	0.49	<LOD	<LOD	0.34
Trauma patient	15	1	52	male	8	12	21	2	0.11	12	93	1085	31727	1725	673	2194	992	4750	8.44	8.26	0.22	0.25	0.41	<LOD	0.39	0.14	<LOD	0.15	1.10	<LOD	1.39	0.36	<LOD	1.54	<LOD	<LOD	<LOD	<LOD	<LOD	<LOD	0.07	0.66	5.17	1.76	0.42	1.88	<LOD	0.43	0.55
Trauma patient	16	1	70	male	2	11	20	2	0.04	-777	-777	-777	11252	1030	292	931	353	1220	16.8	8.77	0.21	0.25	0.34	<LOD	0.37	0.23	<LOD	0.05	<LOD	<LOD	0.16	<LOD	<LOD	0.23	0.19	<LOD	<LOD	<LOD	<LOD	0.08	<LOD	0.27	2.11	1.58	0.17	0.51	<LOD	0.28	0.27
Trauma patient	20	1	68	male	19	19	22	2	0.02	81	14	974	20693	1606	415	1058	340	1119	7.47	11.1	0.17	0.18	0.29	<LOD	0.33	0.33	<LOD	<LOD	<LOD	<LOD	0.26	<LOD	0.07	0.01	<LOD	<LOD	<LOD	<LOD	<LOD	<LOD	<LOD	0.46	3.69	0.98	0.63	0.41	<LOD	0.26	0.25
Trauma patient	21	1	21	male	2	15	19	2	0.05	326	87	2468	27841	1669	488	1475	366	1196	5.18	6.23	0.24	0.25	0.47	0.19	0.54	0.32	0.13	0.09	0.84	<LOD	0.14	0.63	0.08	1.89	<LOD	<LOD	<LOD	<LOD	0.10	<LOD	<LOD	0.45	5.71	1.41	0.44	0.45	<LOD	0.28	0.36
Trauma patient	23	1	29	female	2	26	22	2	0.02	71	32	1063	46712	3275	668	2445	410	2463	9.21	13.4	0.17	0.18	0.35	0.18	0.36	0.28	0.12	<LOD	<LOD	<LOD	0.09	0.50	0.07	1.52	<LOD	<LOD	<LOD	<LOD	<LOD	<LOD	<LOD	0.68	3.13	1.42	0.28	0.48	<LOD	0.28	0.34
Trauma patient	24	1	68	male	11	14	17	2	0.11	827	155	2706	37214	2651	741	3292	479	2513	7.31	7.00	0.24	0.26	0.45	0.19	0.51	0.39	0.16	0.14	0.87	1.20	0.15	<LOD	<LOD	0.14	<LOD	<LOD	<LOD	<LOD	<LOD	0.03	<LOD	0.98	12.1	2.59	1.04	0.80	<LOD	0.35	0.32
Trauma patient	27	1	44	male	2	13	19	2	-777	248	30	2417	42016	4457	933	4033	709	4274	5.79	8.85	0.24	0.20	0.40	<LOD	0.47	0.29	0.12	<LOD	<LOD	1.56	0.35	1.11	0.10	3.22	<LOD	<LOD	<LOD	<LOD	0.08	0.06	<LOD	1.31	12.0	4.35	0.81	1.37	<LOD	0.38	0.60
[illegible]	[illegible]	[illegible]	[illegible]	[illegible]	[illegible]	[illegible]	[illegible]	[illegible]	[illegible]	[illegible]	[illegible]	[illegible]	[illegible]	[illegible]	[illegible]	[illegible]	[illegible]	[illegible]	[illegible]	[illegible]	[illegible]	[illegible]	[illegible]	[illegible]	[illegible]	[illegible]	[illegible]	[illegible]	[illegible]	[illegible]	[illegible]	[illegible]	[illegible]	[illegible]	[illegible]	[illegible]	[illegible]	[illegible]	[illegible]	[illegible]	[illegible]	[illegible]	[illegible]	[illegible]	[illegible]	[illegible]	[illegible]	[illegible]

ID identification number, ICU intensive care unit, ISS injury severity score, <LOD not detected, below limit of detection, -777 sample/patient not available, n.m. not measured; n.a. not applicable
